# Health-promoting phytochemicals and antioxidant capacity in different organs from six varieties of Chinese kale

**DOI:** 10.1038/s41598-019-56671-w

**Published:** 2019-12-30

**Authors:** Jiaqi Chang, Mengyu Wang, Yue Jian, Fen Zhang, Jun Zhu, Qiaomei Wang, Bo Sun

**Affiliations:** 1 Key Laboratory of Horticultural Plant Growth, Development and Quality Improvement, Department of Horticulture, Zhejiang University, Hangzhou, 310058 China; 2 College of Horticulture, Sichuan Agricultural University, Chengdu, 611130 China; 3 Institute of Bioinformatics, Zhejiang University, Hangzhou, 310058 China

**Keywords:** Agricultural genetics, Plant physiology

## Abstract

Chinese kale (*Brassica oleracea* var. *alboglabra*) has high nutritional value. This study investigated the contents of glucosinolates, antioxidants (chlorophylls, carotenoids, vitamin C, and total phenolics), and antioxidant capacity in five organs from six varieties of Chinese kale. The highest concentrations of individual and total glucosinolates were in the roots and inflorescences, respectively. The highest levels of antioxidants and antioxidant capacity were in inflorescences and leaves. Plant organs played a predominant role in glucosinolate and antioxidant accumulation. Glucoiberin, glucoraphanin, and glucobrassicin, the main anticarcinogenic glucosinolates, could be enhanced simultaneously because of their high positive correlations. The relationship between glucosinolates and antioxidant capacity indicated that glucobrassicin might contribute to the total antioxidant capacity. These results provide useful information related to consumption, breeding of functional varieties, and use of the non-edible organs of Chinese kale.

## Introduction

Chinese kale (*Brassica oleracea* var. *alboglabra*) (Brassicaceae) is widely distributed in southern China and Southeast Asia. It is usually grown for its bolting stems, which are consumed. Chinese kale is highly nutritious because of its health-promoting phytochemicals, including glucosinolates, carotenoids, vitamin C, and phenolic compounds^1–3^. Glucosinolates are sulfur- and nitrogen-containing secondary metabolites that exist mainly in the Brassicaceae^4^. They are grouped into aliphatic, aromatic, and indole glucosinolates^5,6^. Glucosinolates and their degradation products have miscellaneous biological functions. Isothiocyanates, glucosinolate hydrolysis products, have significant anticarcinogenic activity. Isothiocyanates possess protective effects against different types of cancer, particularly bladder, colon, and lung cancer^7^.Carotenoids, efficient quenchers of singlet oxygen, can scavenge free radicals and thus prevent the development of cancer^8^. Vitamin C can scavenge superoxide and hydroxyl radicals and act as a chain-breaking antioxidant in lipid peroxidation^9,10^. Phenolics have biological effects, such as inhibition of low-density lipoprotein oxidation, and antimicrobial and anticarcinogenic activity^11^.

The compositions and levels of phytochemicals in plants can be influenced by genotype and environmental factors. These include plant variety, organ, developmental stage, drought stress, and insect feeding^6,12–14^. Variation of glucosinolates and other nutrients in edible parts of Chinese kale varieties has been previously described^2,15,16^. However, a description of the profile and content of glucosinolates and antioxidants among plant organs is unavailable. Therefore, the objective of this study was to evaluate the variation of the composition and contents of glucosinolates and antioxidants in different organs of typical varieties of Chinese kale, and analyze the genetic effects and correlation. The results will reveal the nutritional characteristics of these different varieties, provide consumer information on consumption of edible organs, and offer ideas about possible uses for the non-edible organs.

## Results

### Contents of the main health-promoting phytochemicals

Glucosinolates, four main antioxidants (chlorophylls, carotenoids, vitamin C, and total phenolics), and antioxidant capacity were detected in all of the Chinese kale samples (Table 1). Thirteen glucosiolates were identified. Nine of these were detected in all of the varieties and organs. Glucoiberin, glucoerucin, 4-hydroxyglucobrassicin, and gluconasturtiin were not detected in some samples. The total glucosinolate content ranged from 2.83 to 50.65 μmol g^−1^ DW, with an average value of 18.93 μmol g^−1^ DW (Table 1). Gluconapin was one of the most abundant glucosinolates in all of the varieties while glucoiberin, glucoalyssin, gluconapolelferin, and 4-hydroxyglucobrassicin had low concentrations in some varieties and organs. Carotenoid content in samples ranged from 0.23 to 33.93 mg·100 g^−1^ FW. The ratio between the highest and the lowest content between samples was as high as 150. The difference in the contents of total phenolics was small, but the ratio of the highest and the lowest content reached a maximum of 2.34-fold.

**Table 1:** Mean and range of glucosinolates and antioxidants in Chinese kale among different organs and varieties.

Chemical constituents	Abbreviation	Range	Mean	Median
Total glucosinolates (μmol g^−1^ DW)	TG	2.83–50.65	18.93	10.97
***Aliphatic*** **glucosinolates**				
Glucoiberin	GIB	0.00–0.64	0.14	0.07
Progoitrin	PRO	0.01–2.33	0.61	0.28
Sinigrin	SIN	0.03–5.58	1.19	0.60
Glucoraphanin	GRA	0.04–2.97	0.75	0.58
Glucoalyssin	GAL	0.02–0.42	0.12	0.07
Gluconapin	GNA	0.99–34.79	8.60	6.50
Glucoerucin	GER	0.00–7.92	1.43	0.20
Gluconapolelferin	GNL	0.01–0.15	0.05	0.04
Total aliphatic glucosinolates	TALG	1.79–40.79	12.89	9.06
***Indole*** **glucosinolates**				
4-Hydroxyglucobrassicin	4-OHGBS	0.00–0.66	0.14	0.04
Glucobrassicin	GBS	0.22–8.54	1.71	0.99
4-Methoxyglucobrassicin	4-OMGBS	0.05–1.39	0.31	0.18
Neoglucobrassicin	NGBS	0.03–3.01	0.79	0.48
Total indole glucosinolates	TIG	0.32–10.27	2.95	1.97
***Aromatic*** **glucosinolates**				
Gluconasturtiin	GST	0.00–26.08	3.09	0.09
Total aromatic glucosinolates	TARG	0.00–26.08	3.09	0.09
Chlorophylls (mg·100 g^−1^ FW)	Chl	4.85–212.33	55.81	20.66
Carotenoids (mg·100 g^−1^ FW)	Car	0.23–33.93	9.76	4.61
Vitamin C (mg·100 g^−1^ FW)	Vc	39.97–155.82	100.61	97.07
Total phenolics (mg GAE g^−1^ DW)	TP	3.75–8.79	6.67	6.82
Antioxidant capacities (μmol g^−1^ DW)	AC	28.54–122.18	78.61	83.29

### Variance analysis of genetic effects

Almost all of the ratios of variance on glucosinolates were significant at the 0.01 or 0.05 levels, yet the respective proportions were distinct (Table 2). The ratios of organ variance on glucosinolates were higher than the variety and interaction (variety × organ) variances. With the exceptions of glucoiberin and sinigrin, the ratios of organ variance on glucosinolates all exceeded 50%. The ratio of organ variance for glucoerucin reached 93.0% (Table 2), and the content of glucoerucin was mainly influenced by organ levels. The interaction variance values on glucosinolates surpassed the corresponding variety variance levels, except for glucoiberin, glucoraphanin, and glucobrassicin. For example, the ratio of the interaction variance on 4-hydroxyglucobrassicin was 34.3%, which was exceeded only by the organ variance (50.9%).

**Table 2:** Estimated proportions of variance components for glucosinolates in Chinese kale.

Parameter	*V*_*V*_/*V*_*P*_^a^	*V*_*O*_/*V*_*P*_^b^	*V*_*OV*_/*V*_*P*_^c^	*V*_*e*_/*V*_*P*_^d^
Glucoiberin	0.302**^e^	0.378**	0.263**	0.057**
Progoitrin	0.108**	0.580**	0.271**	0.040**
Sinigrin	0.225**	0.457**	0.301**	0.017**
Glucoraphanin	0.230**	0.574**	0.151**	0.045**
Glucoalyssin	0.044**	0.671**	0.188**	0.097**
Gluconapin	0.103**	0.668**	0.203**	0.026**
Glucoerucin	0.005**	0.930**	0.050**	0.014
Gluconapolelferin	0.147**	0.587**	0.207**	0.059**
4-Hydroxyglucobrassicin	0.109**	0.509**	0.343**	0.039**
Glucobrassicin	0.242**	0.503**	0.228**	0.027*
4-Methoxyglucobrassicin	0.021**	0.800**	0.154**	0.024*
Neoglucobrassicin	0.124**	0.555**	0.295**	0.026*
Gluconasturtiin	0.001	0.819**	0.170**	0.011
Total aliphatic glucosinolates	0.071**	0.781**	0.121**	0.026**
Total indole glucosinolates	0.198**	0.537**	0.230**	0.035**
Total aromatic glucosinolates	0.001	0.819**	0.170**	0.011
Total glucosinolates	0.010*	0.857**	0.111**	0.023*

^a^*V*_*V*_/*V*_*P*_ = ratio of variety variance to phenotypic variance.

^b^*V*_*O*_/*V*_*P*_ = ratio of organ variance to phenotypic variance.

^c^*V*_*VO*_/ *V*_*P*_ = ratio of variety × organ interaction variance to phenotypic variance.

^d^*V*_*e*_/*V*_*P*_ = ratio of error variance to phenotypic variance.

^e^* and ** indicate significance at 0.05 and 0.01 probability levels, respectively.

The ratio pattern of variance on antioxidant contents and antioxidant capacity was similar to the glucosinolates (Table 3). Specifically, the ratios of organ variance on all of the antioxidants as well as antioxidant capacity surpassed those of variety and interaction variances. The ratios of organ variance on chlorophylls, carotenoids and vitamin C were extremely high and the levels were all >90%. The ratios of organ variance on total phenolics and antioxidant capacity were lower but the values still exceeded 60%. These results demonstrated that organ effects played major roles in the variation of the antioxidant contents and antioxidant capacity. The results also showed that the total phenolics content and antioxidant capacity was influenced by interaction effects (Table 3).

**Table 3:** Estimated proportions of variance components for antioxidants in Chinese kale.

Parameter	*V*_*V*_/*V*_*P*_^a^	*V*_*O*_/*V*_*P*_^b^	*V*_*OV*_/*V*_*P*_^c^	*V*_*e*_/*V*_*P*_^d^
Chlorophylls	0.007**^e^	0.952**	0.030**	0.011
Carotenoids	0.000	0.965**	0.004	0.030
Vitamin C	0.019*	0.925**	0.035**	0.021
Total phenolics	0.11**	0.646**	0.212**	0.032**
Antioxidant capacities	0.03**	0.661**	0.285**	0.024*

^a^*V*_*V*_/*V*_*P*_ = ratio of variety variance to phenotypic variance.

^b^*V*_*O*_/*V*_*P*_ = ratio of organ variance to phenotypic variance.

^c^*V*_*VO*_/ *V*_*P*_ = ratio of variety × organ interaction variance to phenotypic variance.

^d^*V*_*e*_/*V*_*P*_ = ratio of error variance to phenotypic variance.

^e^* and ** indicate significance at 0.05 and 0.01 probability levels, respectively.

### Prediction of genetic effects

Genetic effects were further analyzed. The organ effects of inflorescences and roots were mostly positive, whereas organ effects of bolting stems, leaves, and petioles were mostly negative for both total and individual glucosinolates (Table 4). Most of the organ effects were significant. Estimates of organ effects showed that the inflorescence was better than other organs for improved glucoiberin, sinigrin, glucoraphanin, glucoalyssin, gluconapin, glucobrassicin, as well as total aliphatic and total indole glucosinolates. Roots could significantly increase the contents of other glucosinolates (progoitrin, glucoerucin, gluconapolelferin, 4-hydroxyglucobrassicin, 4-methoxyglucobrassicin, neoglucobrassicin, gluconasturtiin, total aromatic, and total glucosinolates). In contrast, the lowest levels of organ effects on glucoiberin, progoitrin, glucoraphanin, glucoerucin, 4-methoxyglucobrassicin, neoglucobrassicin, gluconasturtiin, and total aromatic glucosinolates were detected in leaves, and the lowest levels on other glucosinolate traits were observed in petioles (Table 4). The value of organ effects of inflorescence on gluconapin was very high (11.415). Similarly, the levels of roots on gluconasturtiin and glucoerucin were 11.847 and 4.841, respectively, and surpassed those of other organs. The predication values of organ effects on the main antioxidants and antioxidant capacity are displayed in Table 5. The highest predication values for chlorophylls, carotenoids, and antioxidant capacity were in leaves, and the highest levels for vitamin C and total phenolics were in inflorescences. The lowest levels for chlorophylls and vitamin C were in petioles and the lowest values for total phenolics and antioxidant capacity were in roots (Table 5).

**Table 4:** Predication values of organ effects on glucosinolates/(μmol g^−1^ DW) in Chinese kale.

Name	Inflorescences	Bolting stems	Leaves	Petioles	Roots
Glucoiberin	0.189**^a^	−0.010**	−0.101**	−0.069**	−0.009**
Progoitrin	0.501**	−0.259**	−0.483**	−0.466**	0.708**
Sinigrin	1.713**	−0.198**	−0.772**	−0.858**	0.115**
Glucoraphanin	1.036**	−0.139**	−0.509**	−0.348**	−0.040**
Glucoalyssin	0.144**	−0.061**	−0.061**	−0.066**	0.043**
Gluconapin	11.415**	−2.689**	−5.339**	−6.151**	2.764**
Glucoerucin	−1.102**	−1.004**	−1.399**	−1.337**	4.841**
Gluconapolelferin	0.014**	−0.016**	−0.021**	−0.027**	0.051**
4-Hydroxyglucobrassicin	0.046**	−0.038**	−0.120**	−0.120**	0.232**
Glucobrassicin	2.464**	−0.826**		−0.953**	−0.570**
4-Methoxyglucobrassicin	−0.136**	−0.048**	−0.216**	−0.158**	0.557**
Neoglucobrassicin	0.188*	−0.201**	−0.624**	−0.448**	1.085**
Gluconasturtiin	−2.910**	−2.927**	−3.016**	−2.994**	11.847**
Total aliphatic glucosinolates	14.160**	−4.419**	−8.794**	−9.439**	8.491**
Total indole glucosinolates	2.572**	−1.116**	−1.077**	−1.683**	1.303**
Total aromatic glucosinolates	−2.910**	−2.927**	−3.016**	−2.994**	11.847**
Total glucosinolates	13.898**	−8.518**	−12.951**	−14.197**	21.768**

^a^* and ** indicate significance at 0.05 and 0.01 probability levels, respectively.

Empty cells represent no significant difference in data. The same below.

**Table 5:** Predication values of organ effects on main antioxidants and antioxidant capacities in Chinese kale.

Organ	Chlorophylls (mg·100 g^−1^ FW)	Carotenoids (mg·100 g^−1^ FW)	Vitamin C (mg·100 g^−1^ FW)	Total phenolics (mg GAE g^−1^ DW)	Antioxidant capacities (μmol g^−1^ DW)
Inflorescences	−17.770**^a^	−1.103**	42.434**	1.386**	20.686**
Bolting stems	−46.700**	−8.942**	−11.864**	−1.081**	−20.531**
Leaves	112.099**	18.607**	17.333**	0.964**	25.224**
Petioles	−47.629**	−8.562**	−47.903**		
Roots				−1.340**	−26.366**

^a^*and **indicate significance at 0.05 and 0.01 probability levels, respectively.

In addition to organ effects, nine interaction effects, the corresponding variance ratios of which exceeded 20%, were estimated to determine the changes of glucosinolate concentrations among varieties and organs. The predicated interaction effect levels were quite different (Table 6). For example, the predicated values for gluconapin and neoglucobrassicin were 13.420 and 1.788 in DFZC inflorescences, respectively. However, the lowest levels for glucoiberin and sinigrin were also detected in the same sample. These results suggest that the distinct objectives will be best achieved by selecting suitable combinations between variety and organ.

**Table 6:** Predication values of variety × organ interaction effects on glucosinolates/(μmol g^−1^ DW) in Chinese kale.

Variety	Organ	GIB	PRO	SIN	GNA	GNL	4-OHGBS	GBS	NGBS	TIG
ZHSN	Inflorescences	−0.175** ^a^	−0.319**	−1.322**		−0.020**		−0.962**	−0.396**	−1.347**
	Bolting stems				−1.576*			0.281**	0.198**	0.468**
	Leaves	0.053**	0.123**	0.352**			0.036**	−0.782**	0.333**	
	Petioles	0.040**	0.118**	0.437**	0.336*	0.006*	0.037**	0.174**	0.082**	0.339**
	Roots		−0.144*				−0.165**	0.588**	−0.783**	−0.502**
SJCT	Inflorescences		−0.480**	2.336**	7.862**	0.021**	−0.083**	−0.588**	−0.773**	−1.463**
	Bolting stems			0.543*	−1.688*			0.434**		0.390*
	Leaves	−0.055**	−0.179*	−0.930**	−5.058**		−0.056**	−0.869**	−0.174*	−1.169**
	Petioles	−0.056**		−0.883**	−4.519**	−0.017**	−0.056**	0.058*		
	Roots		0.969**		7.103**		0.285**		0.966**	1.413**
CHDR	Inflorescences	0.286**	1.158**	2.142**		−0.015**	0.098*	0.836*	−0.451**	
	Bolting stems				−3.007**	−0.017**			0.079*	
	Leaves		−0.316**	−0.435**		−0.015**	−0.088**	−0.733**	−0.125*	−1.005**
	Petioles	−0.096**	−0.382**	−0.619**			−0.097**			−0.367*
	Roots	−0.048*			2.042**	0.054**	0.205**		0.434**	0.645**
DFZC	Inflorescences	−0.220**	0.713**	−1.913**	13.420**	0.050**			1.788**	2.409**
	Bolting stems		−0.202*			0.013*	0.021*		−0.223**	
	Leaves	0.067**	−0.263**	0.462**		−0.028**	0.055**		−0.678**	−0.806**
	Petioles	0.038**	−0.204**	0.542**	−1.712**		0.072**		−0.324**	
	Roots			−0.346**	−3.934**		−0.289**	−0.481**		−0.871**
FZHH	Inflorescences	0.099**		0.938**	−3.005*	−0.012*	0.110*	−0.709**	−0.356**	−1.095**
	Bolting stems							0.169*		
	Leaves				0.417*	0.016**	−0.128**			
	Petioles	−0.055**		−0.491**		−0.012**	−0.129**	0.493**		0.187*
	Roots	−0.039*					0.252**	−0.480**		
JL-01B	Inflorescences	0.116*	−0.579**	−0.414*	−6.280**		−0.109**	3.290**	0.380*	3.538**
	Bolting stems			−0.202*	4.328**		−0.038**	−1.517**	−0.116*	−1.805**
	Leaves	−0.105*	0.160**		0.563**	0.006**	0.058**	2.247**		2.300**
	Petioles	0.059**	0.191**	0.130**	1.283**	0.005**	0.049**	−1.364**		−1.404**
	Roots	0.115**	−0.266*	0.307*	−4.070**	−0.035**	−0.049*			

^a^* and ** indicate significance at 0.05 and 0.01 probability levels, respectively.

The predication values of variety effects for glucosinolates possessed more than 20% phenotypic variance (Table 7). The contents of four glucosinolates occurred unevenly among the different varieties. JL-01B had the highest values of glucoiberin, glucoraphanin, and glucobrassicin, while the peak level of sinigrin and bottom level of glucobrassicin was found in SJCT.

**Table 7:** Predication values of variety effects on glucosinolates/(μmol g^−1^ DW) in Chinese kale.

Variety	Glucoiberin	Sinigrin	Glucoraphanin	Glucobrassicin
	GIB	SIN	GRA	GBS
ZHSN	−0.096**^a^	−0.657**	−0.180**	−0.546**
SJCT		0.894**	−0.198**	−0.710**
CHDR	0.083**	0.574**		
DFZC	−0.127**	−0.968**	0.457**	−0.357**
FZHH		0.354**	−0.484**	−0.253**
JL-01B	0.136**	−0.197**	0.471**	1.951**

^a^* and ** indicate significance at 0.05 and 0.01 probability levels, respectively.

### Analysis of genetic correlation

The phenotypic and genotypic correlation coefficients between pairs of individual and total glucosinolates were significant at the 0.05 level (Table 8) and most traits were positively correlated. In addition, the values of phenotypic correlation were similar to genotypic correlation values. The highest correlation coefficient was found between gluconasturtiin and total aromatic glucosinolates. This was followed by those of the pairs between gluconapin and total aliphatic glucosinolates, as well as the pairs between total aliphatic glucosinolates and total glucosinolates. The coefficients of these were all >90%. Besides these pairs, the correlation coefficients between pairs of glucoerucin, 4-methoxyglucobrassicin, gluconasturtiin, and total aromatic glucosinolates, which were richer in roots than in other organs, were also high with values > 80%.

**Table 8:** Correlation coefficient^a^ between pairs of glucosinolates and antioxidant capacities in Chinese kale.

Trait	GIB^b^	PRO	SIN	GRA	GNA	GER	GBS	4-OMGBS	NGBS	GST	TALG	TIG	TARG	TG	AC
GIB		0.290**^c^	0.794**	0.543**	0.322**	−0.076**	0.653**		0.119**		0.391**	0.592**		0.343**	
		0.307**	0.831**	0.546**	0.336**	−0.083**	0.674**		0.119**		0.406**	0.612**		0.354**	
PRO	0.729**		0.456**	0.439**	0.739**	0.586**	0.174**	0.519**	0.720**	0.503**	0.840**	0.528**	0.503**	0.844**	−0.196**
			0.460**	0.442**	0.735**	0.588**	0.176**	0.525**	0.728**	0.509**	0.840**	0.536**	0.509**	0.847**	−0.217**
SIN	1.000**	0.817**		0.351**	0.560**		0.422**	0.151**	0.074**	0.112**	0.611**	0.425**	0.112**	0.517**	0.097**
				0.363**	0.562**		0.422**	0.145**	0.072**	0.110**	0.614**	0.425**	0.110**	0.517**	0.097**
GRA	1.000**	0.727**	1.000**		0.687**		0.728**		0.482**	−0.071**	0.661**	0.754**	−0.071**	0.522**	
					0.696**		0.749**		0.486**	−0.074**	0.668**	0.775**	−0.074**	0.527**	
GNA	1.000**	0.874**	1.000**	1.000**		0.222**	0.39**	0.204**	0.542**	0.174**	0.961**	0.570**	0.174**	0.797**	
						0.215**	0.393**	0.198**	0.542**	0.170**	0.96**	0.574**	0.170**	0.795**	
GER		0.763**	0.106**		0.255**		−0.172**	0.847**	0.688**	0.887**	0.438**	0.283**	0.887**	0.696**	−0.496**
							−0.179**	0.852**	0.692**	0.891**	0.434**	0.281**	0.891**	0.695**	−0.512**
GBS	0.958**	0.519**	0.981**	0.929**	0.924**	−0.223**		−0.15**	0.208**	−0.169**	0.373**	0.849**	−0.169**	0.301**	0.23**
								−0.165**	0.200**	−0.174**	0.375**	0.847**	−0.174**	0.298**	0.226**
4-OMGBS		0.767**	0.112**		0.247**	1.000**	−0.261**		0.692**	0.952**	0.400**	0.339**	0.952**	0.704**	−0.556**
									0.693**	0.957**	0.398**	0.330**	0.957**	0.705**	−0.580**
NGBS	0.489**	0.974**	0.582**	0.402**	0.611**	0.965**	0.127*	0.965**		0.635**	0.652**	0.670**	0.635**	0.791**	−0.377**
										0.642**	0.654**	0.667**	0.642**	0.795**	−0.402**
GST		0.754**			0.217**	1.000**	−0.232**	0.988**	0.940**		0.378**	0.297**	1.000**	0.703**	−0.481**
											0.376**	0.298**	1.000**	0.704**	−0.496**
TALG	0.938**	0.978**	0.974**	0.899**	0.972**	0.482**	0.765**	0.483**	0.803**	0.455**		0.636**	0.378**	0.914**	
												0.641**	0.376**	0.913**	
TIG	0.956**	0.970**	1.000**	0.899**	1.000**	0.438**	0.786**	0.402**	0.717**	0.411**	1.000**		0.297**	0.689**	
													0.298**	0.691**	−0.075*
TARG		0.754**			0.217**	1.000**	−0.232**	0.988**	0.940**	1.000**	0.455**	0.411**		0.703**	−0.481**
														0.704**	−0.496**
TG	0.697**	1.000**	0.770**	0.661**	0.825**	0.764**	0.487**	0.748**	0.970**	0.736**	0.938**	0.939**	0.736**		−0.248**
															−0.266**
AC	0.164**	−0.289**	0.178**	0.231**	0.140**	−0.651**	0.632**	−0.744**	−0.591**	−0.631**		0.093*	−0.631**	−0.277**	

^a^The phenotypic correlation coefficient (*r*_*P*_) and genotypic correlation coefficient (*r*_*G*_) were in the upper and lower line in the upper right corner of the table, respectively. The organ correlation coefficient (*r*_*O*_) was in the lower left corner of the table.

^b^The abbreviations in table were shown in Table 1.

^c^* and ** indicate significance at 0.05 and 0.01 probability levels, respectively.

The organ correlation coefficients between pairs of individual and total glucosinolates were positive. All of the negative correlation coefficients were found between glucobrassicin and the four glucosinolates common in roots (glucoerucin, 4-methoxyglucobrassicin, gluconasturtiin, and total aromatic glucosinolates). Similar to phenotypic and genotypic correlation coefficients, the organ correlation coefficients between pairs of the four above-mentioned glucosinolates were also high and the levels were close to 100%. The organ correlation coefficients between pairs of the three glucosinolates with high anticarcinogenic activity (glucoiberin, glucoraphanin, and glucobrassicin) were all >90%.

Estimates of the genetic correlation components between glucosinolate and antioxidant capacity are presented in Table 8. Positive and significant phenotypic and genotypic correlations were observed for glucobrassicin and sinigrin, and the values were <30% (Table 8). However, the organ correlation results suggested that six glucosinolates (glucoiberin, sinigrin, glucoraphanin, gluconapin, glucobrassicin, and total indole glucosinolates) had positive and significant correlations with antioxidant capacity. The level between glucobrassicin and antioxidant capacity was >60%.

The correlation coefficients among pairs of main antioxidants and antioxidant capacity were dramatically significant at the 0.01 level (Table 9). All of the traits were positively correlated to each other. The correlation coefficient of chlorophylls and carotenoids was higher than others, with a phenotypic correlation of 0.982 and a genotypic correlation of 0.989; followed by the correlation coefficient between total phenolics and antioxidant capacity with the phenotypic correlation of 0.781 and a genotypic correlation of 0.819. Similar results were also observed for the two above-mentioned pairs for the organ correlation coefficient (Table 9).

**Table 9:** Correlation coefficient^a^ between pairs of main antioxidants and antioxidant capacities in Chinese kale.

Trait	Chlorophylls	Carotenoids	Vitamin C	Total phenolics	Antioxidant capacities
Chlorophylls		0.982**^b^	0.424**	0.395**	0.551**
		0.989**	0.428**	0.412**	0.564**
Carotenoids	0.996**		0.499**	0.472**	0.604**
			0.506**	0.494**	0.620**
Vitamin C	0.456**	0.528**		0.509**	0.476**
				0.520**	0.483**
Total phenolics	0.557**	0.632**	0.691**		0.781**
					0.819**
Antioxidant capacities	0.758**	0.812**	0.651**	0.997**	

^a^The phenotypic correlation coefficient (*r*_*P*_) and genotypic correlation coefficient (*r*_*G*_) were in the upper and lower line in the upper right corner of the table, respectively. The organ correlation coefficient (*r*_*O*_) was in the lower left corner of the table.

^b^* and ** indicate significance at 0.05 and 0.01 probability levels, respectively.

## Discussion

Glucosinolates and antioxidants commonly accumulate in the vegetative and reproductive organs of plants^17,18^. We studied the genetic effects and correlations of glucosinolates, main antioxidants and antioxidant capacity among the different organs in varieties of Chinese kale. The results demonstrated that Chinese kale is a rich source of glucosinolates and antioxidants, but the levels in individual organs and varieties varied considerably. Similar results have been reported in broccoli, cauliflower, kale, turnips, and *Arabidopsis*^12,14,17,18^.

The glucosinolates can differ in composition and levels among the different organs of individual plants^17,19,20^. Glucoerucin was the predominant glucosinolate in both seeds and roots of rocket salad, while dimeric 4-mercaptobutyl glucosinolate was the main component in the leaves^20^. Similar results were reported by Petersen *et al*. (2002) in *Arabidopsis*^21^. In the current study, variance analysis indicated that the plant organ played a major role in glucosinolate accumulation. The content of total glucosinolates in Chinese kale was highest in roots, and this value was at least three times as high as the value in other organs except for inflorescences. These patterns were similar to results in other glucosinolate-containing species^17,19,20^. As glucosinolates provide a defense against herbivores and pathogens, the pattern of differences among organs in Chinese kale is consistent with theories on the optimal distribution of defense substances^17^. We found that the levels of 4-methoxyglucobrassicin, glucoerucin, and gluconasturtiin in roots were much higher than levels in other organs. Degradation products of glucoerucin inhibited *Pythium irregulare* oospore germination and *Rhizoctonia solani* soil colonization^22^. In addition, *Arabidopsis* expressing the sorghum CYP79A1 or over-expressing the endogenous CYP79A2 accumulated p-hydroxybenzyl or benzyl glucosinolates, and had increased resistance to the bacterial pathogen *Pseudomonas syringae*^23^. Therefore, the glucosinolate profiles in roots may be a defense adaptation to plant pathogens. Inflorescences, the reproductive organ, had high concentrations of major aliphatic glucosinolates and glucobrassicin. Leaves and petioles, two important vegetative organs of Chinese kale, had low level of glucosinolate. One possible reason is that the nutrients and defense compounds are transported from vegetative organs and accumulate gradually in reproductive organs as plants develop. The concentrations of glucosinolates in the edible bolting stems were maintained at intermediate levels (Table 4). However, these levels are much higher than levels in other vegetables^12,18,24^. These results suggest that, besides the edible part, other organs of Chinese kale, especially the roots and inflorescences, could potentially be used to produce functional foods and biopesticides.

Differences in individual and total glucosinolate contents were found among different varieties of Chinese kale. Genetic variation was an important factor determining the glucosinolate profiles. This suggests that new varieties of Chinese kale with optimal contents of various types of glucosinolates could be developed. Similar effects of genotype on the composition and content of glucosinolates have been observed in broccoli and other Brassica vegetables^2,12,14,18,25^.

Some glucosinolates provide health benefits by reducing the risk of certain cancers. For example, sulforaphane, the isothiocyanate product of glucoraphanin, is a potent inducer of mammalian detoxication and antioxidant (phase 2) enzyme activities that protect against tumorgenesis^26^. Glucobrassicin is the precursor of indole-3-carbinol that, along with sulforaphane, is a potent anticancer compound found in Brasscia vegetables^26–28^. JL-01B, in this study, could also be used for development of a functional variety, because it is rich in anticarcigenic glucosinolates, such as glucoiberin, glucoraphanin, and glucobrassicin. DFZC is also a good candidate because of its high level of glucoraphanin. The correlation results indicated that the above three anticarcigenic glucosinolates could be increased simultaneously in selective breeding of Chinese kale because of their high positive correlations (Table 8). During improvement of anticarcinogenic potency, the positive sensory attributes and nutrient quality of Chinese kale must not be affected by selective breeding. For instance, 2-propenyl isothiocyanate derived from sinigrin is associated with pungency, bitterness, and lachrymatory effects. Progoitrin can have a goitrogenic effect on animals^14,18,29^. However, the high level of positive correlations between the three anticarcinogenic glucosinolates and sinigrin and progoitrin suggest that will be difficult to enhance the anticarcinogenic qualities without deleterious side effects.

Some glucosinolates contribute to the antioxidant capability of the plant. Glucoraphasatin, which is the main glucosinolate in radish sprouts, displayed antioxidant activity and contributed to the total antioxidant capacity of radish sprout extract^30^. Glucoerucin and 4-methoxyglucobrassicin are good antioxidants because of their ability to decompose hydroperoxides and hydrogen peroxide^20,31^. In contrast to anticarcinogenic ability, the relationship between glucosinolates and antioxidant activity is unclear. We analyzed the correlations between glucosinolate and antioxidant capacity (Table 8) and found that glucobrassicin and sinigrin had positive phenotypic correlations with antioxidant capacity. Six glucosinolates (glucoiberin, sinigrin, glucoraphanin, gluconapin, glucobrassicin, and total indole glucosinolates) also had positive organ correlations with antioxidant capacity. The value between glucobrassicin and antioxidant capacity exceeded 60%. The results imply that these kinds of glucosinolates, especially glucobrassicin, are involved in plant antioxidant activity.

Antioxidants are health-promoting phytochemicals in horticultural crops. Carotenoids, vitamin C, total phenolics, and chlorophylls have high antioxidant potential and help provide protection against many types of cancer^8–10,32,33^. The plant antioxidant capacity is reflected by the synergetic effect of multiple antioxidants. Our results indicate that Chinese kale contains high amounts of main antioxidants and has high antioxidant capacity. The high positive correlations between antioxidant capacity and main antioxidants (carotenoids, vitamin C, total phenolics, and chlorophylls) imply that the main antioxidants tested contribute to the total antioxidant capacity in Chinese kale and that they could be improved simultaneously (Table 9). Variance analysis of genetic effects indicated that organ played a predominant role in the contents of main antioxidants and antioxidant capacity as it did in the content of glucosinolates (Table 4). Therefore, it could be easier to select suitable combinations of organs and varieties for utilization of the health-promoting compounds. Although the content of vitamin C in bolting stems was at a low levels among the organs (Table 5), the content was higher than levels in many other vegetables^24,34^.

In conclusion, Chinese kale is a rich source of health-promoting phytochemicals, including glucosinolates and antioxidants. The highest concentrations of individual and total glucosinolates were found in roots and inflorescences, respectively, while the highest levels of main antioxidants (carotenoids, vitamin C, total phenolics, and chlorophylls) and antioxidant capacity occurred in inflorescences and leaves. The non-edible organs (roots, inflorescences, and leaves) have potential for other uses, such as functional foods and biopesticides. JL-01B and DFZC are good candidates for daily consumption and breeding programs since they had high levels of anticarcinogenic glucosinolates in the bolting stems. The genetic effect of organ played a primary role in the accumulation of glucosinolates and antioxidants, while the interaction effects were significant for some compounds. High positive correlations between pairs of anticarcinogenic glucosinolates (glucoiberin, glucoraphanin, and glucobrassicin) and main antioxidants, respectively, indicated that they could be increased simultaneously by selective breeding of Chinese kale varieties. However, it will be difficult to reduce the bitterness that accompanies the enhancement of anticarcinogenic glucosinolates.

## Materials and Methods

### Sample collection and preparation

Six varieties (cv: ZHSN, SJCT, CHDR, DFZC, FZHH, and JL-01B) of Chinese kale were selected. The seeds were sown in trays containing peat and vermiculite (3:1) in a greenhouse at Zhejiang University (Hangzhou, China) with a daily high temperature of 25 °C and a night temperature of 20 °C. Three weeks later, 50 seedlings of each variety with 3–5 true leaves were transplanted into an outdoor field with a row distance of 40 cm and an individual plant distance of 30 cm. Water and fertilizer were applied as necessary.

The whole plants were harvested when the bolting stems with inflorescences were as tall as the apical leaves. This indicates that the edible organ was mature. Plants with equivalent size were selected, and the harvest was conducted in early morning. The plants were placed on ice and transported to the laboratory. For each variety, five plants represented a replicate, and three independent replicates were taken for analysis. The sampled plants were divided into five parts, namely, inflorescences, bolting stems, leaves, petioles, and roots. They were surface-sterilized with a solution of 50 mg kg^−1^ NaClO for 1 min and dried with a hair drier for 1 min. Parts of fresh samples were analyzed for chlorophylls, total carotenoids, and vitamin C. Other parts were frozen, lyophilized in a freeze dryer (VirTis Inc., New York, USA), and stored at −20 °C for analyses of glucosinolates, total phenolics, and antioxidant capacity.

### Glucosinolate composition and contents

Glucosinolates were extracted and analyzed as previously described^2^. Briefly, freeze-dried samples (100 mg) were boiled in 4 ml water for 10 min. The supernatant was collected after centrifugation (5 min, 7000 g), and the residues were washed once with water (4 ml), centrifuged, and then combined with the previous extract. The aqueous extract was applied to a DEAE-Sephadex A-25 (40 mg) column (pyridine acetate form) (GE Healthcare, Piscataway, NJ). The column was washed three times with 1 ml pyridine acetate (20 mM) and twice with 1 ml water. The glucosinolates were converted into their desulpho analogues by overnight treatment with 100 μl of 0.1% (1.4 units) aryl sulphatase (Sigma), and the desulphoglucosinolates were eluted with 2 × 0.5 ml water. HPLC analysis of desulphoglucosinolates was conducted using a Waters HPLC instrument equipped with a Model 2996 PDA absorbance detector (Waters, USA). Samples (20 μl) were separated at 30 °C on a Waters Spherisorb C18 column (250 × 4.6 mm i.d.; 5 μm particle size) using acetonitrile and water at a flow rate of 1.0 ml min^−1^. The procedure employed isocratic elution with 1.5% acetonitrile for the first 5 min; a linear gradient to 20% acetonitrile over the next 15 min followed by isocratic elution with 20% acetonitrile for the final 10 min. Absorbance was detected at 226 nm.

### Determination of chlorophyll contents

Chlorophyll content was analyzed as previously described^35^. Fresh samples (1 g fresh weight (FW)) were ground, extracted with 15 ml 80% acetone and centrifuged at 3000 rpm at room temperature for 10 min. The supernatant was collected and total chlorophyll content was measured by reading the absorbance at 652 nm with a UV-Vis spectrophotometer (UV-2500, Shimadzu Corp., Kyoto, Japan). The chlorophyll content was expressed as mg 100 g^−1^ FW.

### Determination of total carotenoid contents

Total carotenoid content was analyzed as previously described^35^. Samples (1 g, FW) were ground in a mixture of acetone and petroleum ether (1:1, v/v) and extracted twice with the same solution. After washing several times with water, the upper phase was collected and combined as a crude extract. The extracts were brought up to 25 ml with petroleum ether. Total carotenoid content was determined by reading the absorbance at 451 nm with a spectrophotometer. Total carotenoid content was expressed as mg 100 g^−1^ FW.

### Determination of vitamin C contents

Vitamin C content was analyzed as previously described^35^. Frozen samples (3 g) were ground to a fine powder in liquid nitrogen, extracted twice with 10 ml 1.0% (w/v) oxalic acid and centrifuged at 5000 rpm for 5 min. Each sample was filtered through a 0.45-µm cellulose acetate filter. HPLC analysis of vitamin C was conducted using a Waters instrument with a Model 2996 PDA detector (Waters Inc., Milford, USA). Samples (20 µl) were separated at room temperature on a Waters Spherisorb C18 column (250 × 4.6 mm id; 5 µm particle size) using a 0.1% oxalic acid solvent at a flow rate of 1.0 ml min^−1^. The amount of ascorbic acid was calculated from absorbance values at 243 nm, using authentic ascorbic acid as a standard. Results were expressed as mg 100 g^−1^ FW.

### Determination of total phenolic contents

The total phenolics were extracted with 50% ethanol and incubated at room temperature for 24 h in darkness. The suspension was centrifuged at 10,000 rpm for 10 min at room temperature and the supernatant was collected. Phenolic compounds were determined using Folin-Ciocalteu reagent by reading the absorbance at 760 nm as previously described^36^. Gallic acid was used as a standard and the results were expressed as mg gallic acid equivalent (GAE) g^−1^ dry weight (DW).

### Determination of antioxidant capacity

Antioxidant capacity was determined using the Ferric reducing antioxidant power (FRAP) method of Benzie and Strain (1996)^37^. The working FRAP reagent was prepared daily by mixing 300 mM acetate buffer (pH 3.6), 20 mM ferric chloride, and 10 mM 2,4,6-tripyridyl-S-triazine in 40 mM HCl in a ratio of 10:1:1 (v/v/v). The extracts (300 μl) were added to 2.7 ml of the FRAP working solution incubated at 37 °C and vortexed. The absorbance was recorded at 593 nm using a UV-Vis spectrophotometer (UV-2500, Shimadzu Corp., Kyoto, Japan) after the mixture had been incubated at 37 °C for 10 min. FRAP values were calculated from FeSO_4_·7H_2_O standard curves and expressed as µmol g^−1^ DW.

### Statistical analysis

The genetic model developed by Zhu (1996)^38^ was used for the analysis of inheritance. The model used for the analysis is:

$${{\rm{Y}}}_{{\rm{ijk}}}={\rm{\mu }}+{{\rm{G}}}_{{\rm{i}}}+{{\rm{E}}}_{{\rm{j}}}+{{\rm{GE}}}_{{\rm{ij}}}+{{\rm{e}}}_{{\rm{ijk}}},$$

where Y_ijk_ = the phenotypic mean of the cross of variety i and organ j in the kth block; μ = population mean; G_i_ = the variety effect; E_j_ = the organ effect; GE_ij_ = the variety × organ effect; and e_ijk_ = the residual error.

The data were analyzed with TestR Model by MINQUE method for estimating variances and covariances and calculating the ratios of genetic variance over phenotypic variance. All of the data analyses were performed with QGAStation Version 2.0 software. All of the factors were considered as random. The genetic effects were predicted by the adjusted unbiased prediction method (AUP) method^39^, while standard errors of the statistics were obtained by the Jackknife resampling method and Student’s *t*-tests were performed for testing the significance of the obtained parameters^40^.

## References

[1] Abellán Á, Domínguez-Perles R, Moreno DA, García-Viguera C (2019). Sorting out the value of cruciferous sprouts as sources of bioactive compounds for nutrition and health. Nutrients.

[2] Sun B, Liu N, Zhao YT, Yan HZ, Wang QM (2011). Variation of glucosinolates in three edible parts of Chinese kale (*Brassica alboglabra* Bailey) varieties. Food Chem..

[3] Sun B, Yan HZ, Liu N, Wei J, Wang QM (2012). Effect of 1-MCP treatment on postharvest quality characters, antioxidants and glucosinolates of Chinese kale. Food Chem..

[4] Hansen M, Møller P, Sørensen H, Cantwell M (1995). Glucosinolates in broccoli stored under controlled atmosphere. J. Am. Soc. Hortic. Sci..

[5] Halkier BA, Du LC (1997). The biosynthesis of glucosinolates. Trends Plant Sci..

[6] Halkier BA, Gershenzon J (2006). Biology and biochemistry of glucosinolates. Annu. Rev. Plant Biol..

[7] Abdull Razis AF, Noor NM (2015). Naturally-occurring glucosinolates, glucoraphanin and glucoerucin, are antagonists to aryl hydrocarbon receptor as their chemopreventive potency. Asian Pac. J. Cancer P..

[8] Sun T (2018). Carotenoid metabolism in plants: the role of plastids. Mol. Plant.

[9] Travica N (2017). Vitamin C Status and Cognitive Function. A Systematic Review..

[10] Gayosso-García Sancho LE, Yahia EM, González-Aguilar GA (2011). Identification and quantification of phenols, carotenoids, and vitamin C from papaya (*Carica papaya* L., cv. Maradol) fruit determined by HPLC-DAD-MS/MS-ESI. Food Res. Int..

[11] Costea T, Nagy P, Ganea C, Szöllősi J, Mocanu MM (2019). Molecular Mechanisms and Bioavailability of Polyphenols in Prostate Cancer. Int. J. Mol. Sci..

[12] Kushad MM (1999). Variation of glucosinolates in vegetable crops of *Brassica oleracea*. J. Agr. Food Chem..

[13] Fahey JW, Zalcmann AT, Talalay P (2001). The chemical diversity and distribution of glucosinolates and isothiocyanates among plants. Phytochemistry.

[14] Cartea ME, Velasco P, Obregón S, Padilla G, Haro A (2008). Seasonal variation in glucosinolate content in *Brassica oleracea* crops grown in northwestern Spain. Phytochemistry.

[15] Zhang SH (2004). The nutritive value of different strains of cabbage mustard. J. Hebei Normal Univ. Sci. Technol..

[16] Si Y (2009). Analysis on composition and content of glucosinolates in different genotypes of Chinese kale. China Vegetables.

[17] Brown PD, Tokuhisa JG, Reichelt M, Gershenzon J (2003). Variation of glucosinolate accumulation among different organs and developmental stages of *Arabidopsis thaliana*. Phytochemistry.

[18] Padilla G, Cartea ME, Velasco P, Haro A, Ordás A (2007). Variation of glucosinolates in vegetable crops of *Brassica rapa*. Phytochemistry.

[19] Sang JP, Minchinton IR, Johnstone PK, Truscott RJW (1984). Glucosinolate profiles in the seed, root and leaf tissue of cabbage, mustard, rapeseed, radish and swede. Can. J. Plant Sci..

[20] Kim SJ, Ishii G (2006). Glucosinolate profiles in the seeds, leaves and roots of rocket salad (*Eruca satica* Mill.) and anti-oxidative activities of intact plant powder and purified 4-methoxyglucobrassicin. Soil Sci. Plant Nutr..

[21] Petersen BL (2002). Composition and content of glucosinolates in developing *Arabidopsis thaliana*. Planta.

[22] Manici LM (2000). Suppressive activity of some glucosinolate enzyme degradation products on *Pythium irregulare* and *Rhizoctonia solani* in sterile soil. Pest Manag. Sci..

[23] Brader G, Mikkelsen MD, Halkier BA, Palva ET (2006). Altering glucosinolate profiles modulates disease resistance in plants. Plant J..

[24] Volden J, Bengtsson GB, Wichlund T (2009). Glucosinolates, L-ascorbic acid, total phenols, anthocyanins, antioxidant capacities and colour in cauliflower (*Brassica oleracea* L. ssp. *botrytis*); effects of long-term freezer storage. Food Chem..

[25] Yan XF, Chen SX (2007). Regulation of plant glucosinolate metabolism. Planta.

[26] Zhang YS, Talalay P (1994). Anticarcinogenic activities of organic isothiocyanates: chemistry and mechanism. Cancer Res..

[27] Shan YQ, Zhang JL, He HJ (2007). The property research of glucosinolate and sulforaphane in Cruciferae. Food Sci. Technol..

[28] Lou P (2008). Quantitative trait loci for glucosinolate accumulation in *Brassica rapa* leaves. New Phytol..

[29] Van Doorn HE (1998). The glucosinolates sinigrin and progoitrin are important determinants for taste preference and bitterness of Brussels sprouts. J. Sci. Food Agric..

[30] Barillari J (2008). Kaiware Daikon (*Raphanus sativus* L.) extract: a naturally multipotent chemopreventive agent. J. Agr. Food Chem..

[31] Barillari J (2005). Direct antioxidant activity of purified glucoerucin, the dietary secondary metabolite contained in rocket (*Eruca sativa* Mill.) seeds and sprouts. J. Agr. Food Chem..

[32] Lanfer-Marquez UM, Barros RMC, Sinnecker P (2005). Antioxidant activity of chlorophylls and their derivatives. Food Res. Int..

[33] Reddy VK, Sreeramulu C, Raghunath D (2010). M. Antioxidant activity of fresh and dry fruits commonly consumed in India. Food Res. Int..

[34] Bahorun T, Luximon-Ramma A, Crozier A, Aruoma OI (2004). Total phenol, flavonoid, proanthocyanidin and vitamin C levels and antioxidant activities of Mauritian vegetables. J. Sci. Food Agr..

[35] Sun B, Yan HZ, Zhang F, Wang QM (2012). Effects of plant hormones on main health-promoting compounds and antioxidant capacity of Chinese kale. Food Res. Int..

[36] Ainsworth EA, Gillespie KM (2007). Estimation of total phenolic content and other oxidation substrates in plant tissues using Folin-Ciocalteu reagent. Nat. Protoc..

[37] Benzie IF, Strain JJ (1996). The ferric reducing ability of plasma (FRAP) as a measure of “antioxidant power”: the FRAP assay. Anal. Biochem..

[38] Zhu J (1996). Analytic methods for seed models with genotype × environment interactions. Acta Genet. Sin..

[39] Zhu J, Weir BS (1996). Diallel analysis for sex-linked and maternal effects. Theor. Appl. Genet..

[40] Miller RG (1974). The jackknife: a review. Biometrika.

